# Current stroke rehabilitation services and physiotherapy research in South Africa

**DOI:** 10.4102/sajp.v75i1.475

**Published:** 2019-07-22

**Authors:** Mokgobadibe V. Ntsiea

**Affiliations:** 1 Department of Physiotherapy, School of Therapeutic Sciences, Faculty of Health Sciences, University of the Witwatersrand, Johannesburg, South Africa

**Keywords:** stroke, post-discharge rehabilitation service, physiotherapy, South Africa, outcome measures, caregivers

## Abstract

**Background:**

Stroke is one of the most common causes of morbidity and disability in South Africa, with the burden of stroke particularly high in rural South Africa.

**Objectives:**

The aim of this study was to collate South African (SA) physiotherapy stroke rehabilitation research.

**Method:**

A narrative review of physiotherapy stroke rehabilitation research conducted within the last 10 years in South Africa.

**Results:**

Stroke survivors in South Africa have poor functional ability at discharge from the hospital and have poor access to transport, work and education. Their caregivers experience strain and have a poor quality of life. Inpatient rehabilitation services focus on the medical model approach and patients are discharged into family care because of limited rehabilitation facilities. Physiotherapy interventions found to be effective in SA studies: longitudinal shoulder strapping, balance exercises in the community, task-orientated circuit gait training, saccadic eye movement training with visual scanning exercises for unilateral spatial neglect and workplace intervention programmes to increase return to work after stroke. Caregiver education alone and use of pictorial exercise programmes does not improve patients’ functional ability and adherence to home exercise programmes, respectively.

**Conclusion:**

There is a need to focus physiotherapy stroke rehabilitation on barriers that hinder full social integration of the patient, including return to work and improving carer support. Most research reviewed focused on description of the problems experienced; however, more intervention studies are now underway to develop context-specific interventions with feasible treatment intensity, frequencies and equipment requirements. Future research should explore new ways of improving post-discharge rehabilitation services. Examples of intervention research that may be beneficial in a SA context are mirror therapy, mental practice and patient-directed activities in rehabilitation.

**Clinical implications:**

Knowledge of interventions that were found to be effective in this context will encourage clinicians to translate these findings into practice. Noting that outcome measures that are core for stroke rehabilitation are not included in some projects may remind researchers to consider them to make comparisons between different research projects.

## Introduction

Stroke is one of the most common causes of morbidity in South Africa, with an estimated 75 000 strokes occurring in South Africa each year (Bertram et al. 2013). The burden of stroke is particularly high in rural South Africa, with 33 500 strokes occurring in a population of 13 000 000 people in 2011 (Maredza, Bertram & Tollman 2015). Not only is the prevalence of stroke high, but Wolf, Baum and Connor (2009) in a study of 7740 stroke survivors found that 45% of the patients were below the age of 65 and 27% were below the age of 55. The young age of stroke survivors was confirmed by a study conducted within the Gauteng Province by Duff, Ntsiea and Mudzi (2014), which reported a mean age of 51 (s.d. ± 10.5 years) in a population of stroke survivors (*n* = 96) studied. Thus, stroke is no longer considered a disease of older people in South Africa. Stroke has devastating effects on young stroke survivors because they still have more years of social and economic activity than older adults with stroke. Young people living with stroke will arguably need more rehabilitation to enable full integration into the community and work.

Rehabilitation that meets all the needs of a stroke survivor is considered ‘a process of active change by which a person who has become disabled acquires the knowledge and skills needed for optimum physical, psychological and social function’ (British Society of Rehabilitation Medicine 2003:7).

Rehabilitation is thus all encompassing and extends beyond that of addressing body structure and function and activity limitations.

During rehabilitation there is a need to distinguish behavioural restitution (return to pre-stroke function because of neural tissue repair: true recovery without the need to learn compensatory movements for functional ability) and use of compensation strategies (e.g. learning to perform movements using spared muscles and joints without neural repair) (Bernhardt et al. 2017). Behavioural restitution is preferable because of restoration of normal function that does not require learning of compensatory techniques for functional ability. The first week until the first month post-stroke (acute and early subacute) is a critical time to enhance neural plasticity (Krakauer et al. 2012), and thus every effort needs to be made to provide early rehabilitation services to all patients and the focus during this phase should be on restoration and not compensation.

The importance of early post-stroke rehabilitation in a South African (SA) context is highlighted by findings from a study that compared functional outcomes of SA stroke survivors with those of patients in developed countries that tend to focus on early post-stroke rehabilitation. A study by Rhoda et al. (2014) found that functional recovery of stroke survivors in Germany (*n* = 66) was better than that of SA (*n* = 56) patients at 6 months after stroke when assessed using the Rivermead Motor Assessment Scale and the Barthel Index (*p* = 0.0003 and 0.003, respectively). There were no significant differences in age, gender, aphasia and time since stroke onset between the SA and German study sample. All stroke survivors in the German study received rehabilitation from a multidisciplinary team. In the SA study sample, all received physiotherapy and only patients from 16 of the 21 health centres received speech therapy and occupational therapy in addition to physiotherapy. Patients in the SA study were already being seen at community health centres 20 days after stroke when the patients in the German study were still being seen as inpatients at the same time since stroke onset. Thus, the German patients may have received more rehabilitation sessions at this early stage of rehabilitation because they did not have to travel to get to a rehabilitation centre at this stage of their rehabilitation.

Given the information presented, the following questions come to mind: do stroke survivors in South Africa have access to rehabilitation services, and are there feasible intervention strategies suited for resource poor clinical facilities and communities like the ones found in South Africa? Many people undertake stroke rehabilitation research in South Africa, and an overview of this research to inform SA rehabilitation services is thus warranted. Thus, the aims of this review were to make SA stroke rehabilitation research and services visible with the objectives of (1) identifying, critiquing and summarising current SA physiotherapy stroke rehabilitation research, (2) to determine SA physiotherapy stroke rehabilitation research gaps based on the available literature and (3) to establish if there are feasible intervention programmes developed internationally suited for resource poor clinical facilities and communities like those found in South Africa.

## Methods

The literature search for this article was conducted using the following sources: Pubmed, Medline, PEDRO, University of the Witwatersrand, Free State, Cape Town, Western Cape, KwaZulu-Natal, Pretoria, Stellenbosch, Sefako Makgatho Health Sciences library catalogues, Cochrane, Google Scholar and citation tracking. The following search words were used: stroke, physiotherapy, rehabilitation, services, outcome measures and South Africa. Physiotherapy-related studies, studies that included physiotherapy in a multidisciplinary intervention programme and studies that reported general activity and participation restrictions of stroke survivors within the last 10 years were included. Policy documents were included even if they were more than 10 years old provided they were the latest version. The search for stroke in South Africa yielded 839 studies, and when narrowed down to human studies after 2008, it yielded 437 studies that were reduced to 39 studies when limited only to stroke rehabilitation studies. Two independent researchers (V.N. and A.M.) undertook the search for abstracts. Studies identified were read by two independent researchers who applied the following exclusion criteria: non-human subjects and those that did not focus on stroke rehabilitation or services. A third researcher (H.V.) then read all the full-text articles that were identified for inclusion. A quality rating scale was not applied to these studies because the aim was to include all studies irrespective of quality. The quality of the studies was taken into consideration when presenting and discussing results. Data on study methods including specific intervention programmes where applicable were extracted, interpreted and summarised.

### Ethical considerations

The author was not exposed to any patient confidential information and did not have any form of contact with patients or people who participated in the reviewed studies. Articles reviewed are in the public domain and thus the author did not need permission to conduct the review.

## Results

The findings of the review are reported below under the following summary headings: Problems experienced by stroke survivors in South Africa; Problems experienced by caregivers of stroke survivors in South Africa; Stroke rehabilitation services in South Africa; Stroke rehabilitation outcome measures: South African context; Stroke rehabilitation physiotherapy interventions: South African context and General considerations.

### Problems experienced by stroke survivors in South Africa

South African research shows that people following stroke have problems at all levels of the International Classification of Functioning, Disability and Health (ICF). Specific problems identified include both basic and instrumental activities of daily living needed to function within their communities (80.3%) (Rhoda, Mpofu & De Weerdt 2011), dependence with walking and stair climbing at discharge (58% and 78%, respectively) (Joseph & Rhoda 2013) and poor health-related quality of life (79.5%) (Rhoda 2014). Mamabolo et al. (2009) argued that SA stroke survivors have poor quality of life because they are discharged home when they are still functionally dependent (47%) (Mamabolo et al. 2009).

Participation restrictions experienced by SA stroke survivors, especially in poor socio-economic circumstances, include lack of transport and community environmental terrains not conducive to wheelchair use (Rhoda et al. 2015). These restrictions mean that some stroke survivors are not able to attend outpatient rehabilitation services or participate in their communities. Mobility is even more limited for those who are socio-economically disadvantaged as indicated by De Villiers et al. (2011) in their observational study of 196 socio-economically disadvantaged stroke survivors who were followed up for 6 months. These authors measured disability using the modified Rankin Scale and modified Barthel index, and reported that ‘shack^1^’ housing was independently more associated with moderate-to-severe disability (odds ratio 3.42, confidence interval 1.22–9.59, *p* = 0.02) than other forms of housing. Other participation restrictions reported are from an observational cross-sectional study of 97 stroke survivors aged 51 ± 10.5 years within the Gauteng Province, which showed return to work or economic productivity with only 34% of the stroke survivors sampled returning to work 2 years after stroke (Duff et al. 2014). This was similar to a rate of return to work reported in the Eastern Cape Province (*n* = 40; 32%) (Patterson 2018).

### Problems experienced by caregivers of stroke survivors in South Africa

A study conducted at one academic hospital in Gauteng found that patients are discharged less than 2 weeks after stroke and only 7% of stroke survivors had home visits after discharge (Mamabolo et al. 2009). With transport difficulties and thus an inability to attend outpatient rehabilitation services, caregivers appear to carry most of the burden of care. Studies conducted with SA caregivers of stroke survivors (Hilton et al. 2013; Kusambiza-Kiingi, Maleka & Ntsiea 2017) reveal that most caregivers experience strain, with 55% (*n* = 45) and 77% (*n* = 35) scoring more than 7 (severe strain) on the Caregiver Strain Index. Problems of caregivers of patients who have aphasia were compounded by communication problems (Masuku, Mophosho & Tshabalala 2017) with limited access to speech language therapy for advice.

The challenges experienced by stroke survivors and their caregivers are an indication that stroke rehabilitation services should extend beyond the hospital to facilitate recovery and improve quality of life.

### Stroke rehabilitation services in South Africa

There are private and government rehabilitation services in South Africa and both provide services for patients at various phases post-stroke. The phases of stroke recovery are hyper acute (0–24 hours), acute (1–7 days), early subacute (7 days–3 months), late subacute (3–6 months) and chronic stage (> 6 months) (Bernhardt et al. 2017).

Specific stroke management and rehabilitation services vary between facilities depending on availability of resources. However, all health facilities are accredited by the Department of Health which is guided by a number of policies, including the National Rehabilitation Policy of South Africa (2000) and the World Health Organization Community-Based Rehabilitation Guidelines (WHO 2010). These policies recommend creation of the right environment for quality rehabilitation services, improvement in access to rehabilitation services and creation of an environment that enables people with all forms of disability to participate in society. This is still a work in progress as some of these recommendations are still to be met (e.g. not all people have access to full rehabilitation services).

Stroke management in South Africa includes primary prevention. However, after a stroke has occurred, according to the SA guideline for the management of ischaemic stroke, intravenous thrombolytic therapy with recombinant tissue plasminogen activator (tPA) is an accepted therapy within 4.5 h of onset of symptoms, but can only be administered at centres with specific resources (Bryer et al. 2010). Stroke survivors usually start rehabilitation as soon as they become medically stable – ideally during the acute phase (Krakauer et al. 2012). Inpatient rehabilitation services in South Africa generally focus on the medical model approach, which focuses on changes in body structure and function and activity limitations with the hope that this will enable the individual to participate in the community and pays little attention to the barriers that hinder full social integration (Mji et al. 2013). Patients are discharged before functional independence is reached because of a high demand for beds. The average length of stay in tertiary hospitals for a stroke survivor is 6 days (Mudzi, Stewart & Musenge 2012a) and less than 2 weeks in other hospitals (Mamabolo et al. 2009).

When patients are discharged from the hospital, the post-discharge rehabilitation services are not accessible because of transport difficulties and limited community-based rehabilitation service options. Most stroke survivors who receive outpatient rehabilitation at community health centres in South Africa only receive 1–4 h of physiotherapy sessions with a median number of 1.8 h over a 6-month period (Rhoda, Mpofu & De Weerdt 2009).

Some communities have home-based care services, but there is poor referral and articulation with these services (Mabunda, London & Pienaar 2017). According to the South African Framework and Strategy for Disability and Rehabilitation (2015–2020), therapists are supposed to conduct home visits for clients in their homes for specific interventions with follow-up visits by mid-level health workers. It is not clear, however, as to who these proposed mid-level workers are and where they are trained. This ambiguity needs to be addressed because therapists are not coping with the post-discharge rehabilitation needs (Rhoda et al. 2009).

In some instances, as indicated in Mapipa, Wolvaardt and Senkubuge (2016)’s study, patients are given an opportunity to attend outpatient services at local hospitals or primary healthcare clinics, but many do not attend. These authors did one-on-one interviews of 12 stroke survivors who missed their outpatient sessions at a SA public sector hospital in the Pretoria area. Participants gave very positive feedback on the hospital rehabilitation services, but said that fear of losing their jobs and poor transport availability meant that they could not always attend. Participant perceptions were of poor services at the primary healthcare clinics. This is an indication that some patients have opportunities for rehabilitation, but are not able to utilise them fully. Access to stroke rehabilitation services is, however, improving in some communities because of the availability of community service therapists^2^ at community health centres (Mapipa et al. 2016).

More is expected with the transformation of the SA health system towards universal coverage that is meant to overcome the deep inequities, including poor access to rehabilitation services, especially for people who cannot afford private healthcare (Framework and Strategy for Disability and Rehabilitation 2015–2020). Inclusion of stroke units in the SA guidelines for the management of ischaemic stroke (Bryer et al. 2010) is a step in the right direction because treatment in a stroke unit has been shown to increase the likelihood of independence after stroke (Stroke Unit Trialists’ Collaboration 2013). There are government-funded stroke units in the Western Cape and Gauteng and private stroke units in most provinces. Stroke rehabilitation services are expected to be available to more stroke survivors in future with more clearly defined referral pathways and communication between rehabilitation team members (Framework and Strategy for Disability and Rehabilitation 2015–2020).

### Stroke rehabilitation outcome measures: South African context

Measuring outcome following stroke is crucial, but should be relevant and appropriate to the context. For example, challenges that are often not addressed in the literature but are applicable in a SA context are means of administration and time taken to complete the items, the language and rules for translation used, and the cost of outcome measures or licensing and copyright (Joseph & Rhoda 2011).

Assessment and measurement should be done within the ICF framework. The ICF is seen as a framework and classification that provides a common language for all users (World Health Organization 2001). Assessment done within the ICF framework includes body structure and function limitations, and this is important to distinguish true recovery from compensation (Krakauer et al. 2012). To test the effect of interventions on sensorimotor recovery, measurements should be performed within 7 days of stroke onset and repeated up to 3 months after stroke. This is the period when major improvements can be expected. If the aim is to test effect of interventions on ability to compensate, which is typically more than 3 months after stroke, noting stroke severity at stroke onset is strongly recommended (Kwakkel et al. 2017).

The following are recommended as core outcome measures for inclusion in stroke trials (Kwakkel et al. 2017): National Institute of Health Stroke Scale (NIHSS), Fugl-Meyer (FM), Action Research Arm Test (ARAT); 10-m walk test, 6-minute walk test (6MWT); self-report questionnaire of health status (EQ-5D); and simplified modified Rankin Scale (mRS). There was no evidence to support use of any one participation level tool as a core outcome measure (Kwakkel et al. 2017). Outcome measures used in stroke rehabilitation research in South Africa compared to core outcome measure by Kwakkel et al. (2017) are presented in Table 1.

**TABLE 1:** Outcome measures used in stroke rehabilitation research in South Africa compared to core outcome measure recommended by Kwakkel et al. (2017).

Classification	Outcome measures recommended by Kwakkel et al. (2017)	Outcome measures used in South African studies
Classification of stroke severity	National Institute of Health Stroke Scale (NIHSS)	**NHISS** (Recommended by Bryer et al. 2010)
Body function and structure (impairments)	Fugl-Meyer (FM – motor, arm and leg)	Shoulder subluxation finger width measurement system for shoulder subluxation and Ritchie Articular Index for pain (Comley-White et al. 2018)
		Modified Ashworth Scale (MAS) (Comley-White 2015)
		Montreal Cognitive Assessment (MoCA) (Ntsiea et al. 2015)
		Wong-Baker FACES Pain Rating Scale, **Fugl-Meyer Assessment**, Postural Stability Test (Van Wyk 2016)
Activity limitations	Action Research Arm Test (ARAT) for upper limb	Frenchay Arm Test (FAT) (Muller 2015)
		Motor Assessment Scale (Comley-White 2015)
	For walking, 10-m walk test (with gait aids if necessary)	Barthel Index (Mudzi, Stewart & Musenge 2013; Van Wyk, Eksteen & Rheeder 2014)
	A 6-minute walk test (6MWT) is recommended if the primary aim is to measure progress in walking	Functional Independence Measure (FIM) (Muller 2015)
		**6MWT**, Berg Balance Scale (BBS), and Timed Up and Go (TUG) (Knox et al. 2018)
		An eight-camera T-10 Vicon system with Nexus 1.8 software (Vicon Motion System Limited, Oxford, UK) to analyse the 3D kinematics of the trunk during self-selected walking speed (Titus et al. 2018)
		Postural Assessment Scale for Stroke Patients (PASS) (Puckree & Naidoo 2014)
		Rivermead Mobility Index (Mudzi et al. 2013)
		Modified Rivermead Mobility Index (Kara & Ntsiea 2015)
		Frenchay Activities Index and Nottingham Extended Activities of Daily Living Scale (Joseph & Rhoda 2011)
Quality of life	Self-report questionnaire of health status (EQ-5D)	Short Form 36 (Westaway 2010)
		**EQ-5D** (Hilton et al. 2013; Rhoda 2014)
		Caregiver Strain Index (CSI) (Hilton et al. 2013)
		Stroke-Specific Quality of Life Scale (Ntsiea et al. 2015)
Global disability	Simplified modified Rankin Scale (mRS)	**Modified Rankin Scale** (Duff et al. 2014)
		World Health Organisation Disability Assessment Schedule II (WHODAS II) (Arowoiya et al. 2017)
Participation	No evidence to support use of any one tool as core outcome measure	Maleka Stroke Community Reintegration Measure (MSCRIM) (Maleka et al. 2017)
		Reintegration to Normal Living Index (Joseph & Rhoda 2011)
		Stroke Impact Scale 16 (Knox et al. 2018)

Outcome measures used in SA research were identified from individual research papers, electronic dissertations and from a study by Joseph and Rhoda (2011), a systematic review that identified outcome measures used in stroke rehabilitation in South Africa. Most researchers indicated that the outcome measures they used were feasible and applicable to a SA context and some, for example, the Maleka Stroke Community Reintegration Measure (Maleka, Stewart & Hale 2017), were developed specifically for South Africa. Outcome measures that are not freely available, like the Functional Independence Measure, are used mainly in private rehabilitation units for clinical and research purposes. Kwakkel et al.’s (2017) study presents results of a consensus meeting about outcome measures that they suggest should be collected in all stroke recovery trials; these recommendations were aligned with the ICF. All core outcome measures on Kwakkel et al.’s (2017) list were used in more than 20 of the stroke rehabilitation studies in South Africa except for the Action Research Arm test and the 10-m walk test, as shown in Table 1. It would be easier to compare SA stroke research findings from various research settings if at least one of the recommended core outcome measures for every ICF level assessed is included in future research.

It is difficult to know if the outcome measures recommended by Kwakkel et al. (2017) are used in clinical practice in South Africa. A research project to determine this is in progress. While research conditions are not always a true reflection of clinical realities, some outcome measures used in research are applicable in clinical practice and their use in clinical practice should be encouraged. Most patient assessment- and treatment-related records can form part of information or research databases, which can be used to compare patient treatment outcomes, inform practice and policy, and motivate for funds, equipment or rehabilitation space.

Patient demographic and clinical details are required in addition to standardised outcome measures, and thus it is important that all the necessary information is captured for clinical and research records. Kwakkel et al. (2017) recommend capturing of the following demographic and stroke information: age; sex; ethnicity; medical history: vascular risk factors, renal or cardiac failure, prior stroke or transient ischaemic attack, co-morbid conditions; pre-morbid function; education; pre-morbid walking status; pre-morbid living arrangements; stroke severity; active hand movement at stroke onset; ability to walk independently at stroke onset; stroke type; stroke sub-type; stroke location; thrombolysis or reperfusion therapy; and imaging.

Psychological, cognitive, physical, social and lifestyle factors must also be captured. These are recommendations for research, but they can enrich the clinical database of patients’ records as well.

### Stroke rehabilitation physiotherapy interventions: South African context

South African physiotherapy stroke rehabilitation research is undertaken to establish interventions that are context-specific, taking into consideration available resources (human and material), geographical and political factors that are unique to the country or even specific provinces, and we report this research below (Comley-White, Mudzi & Musenge 2018; Kara & Ntsiea 2015; Knox, Stewart & Richards 2018; Mudzi, Stewart & Musenge 2012b; Ntsiea et al. 2015; Puckree & Naidoo 2014; Van Wyk 2016; Van Wyk, Eksteen & Rheeder 2014). We also have included intervention evidence from studies conducted outside South Africa whose findings we consider are relevant to the SA context.

#### Upper limb rehabilitation

Stroke results in upper limb impairments that affect hand and upper limb function, with shoulder subluxation with or without pain often presenting as a complication. A Cochrane systematic review presented moderate quality evidence for upper limb rehabilitation after stroke, establishing the benefits of constraint-induced movement therapy (CIMT), mental practice, mirror therapy, virtual reality, interventions for sensory impairment and a high dose of repetitive task practice (Pollock et al. 2014). In South Africa, there are limited physiotherapy upper limb intervention studies, and thus applicable international literature must be referred to and evidenced-based interventions that can be used in resource poor settings identified for use and possibly modified and evaluated for efficacy in a SA setting. For example, intervention using robotics or those requiring intensive and long one-on-one therapy would not be appropriate in low socio-economic settings. Interventions that may be applicable and require further evaluation include mirror therapy, mental practice, modified CIMT, high-dose repetitive practice and home exercise programmes using items that can be found in most households as equipment.

Shoulder strapping is used in most stroke rehabilitation facilities. Comley-White et al. (2018) did a study in Gauteng to establish the impact of longitudinal strapping when compared to circumferential strapping techniques on patient’s upper limb tone, motor function, pain or subluxation, post-stroke. This was a randomised control trial of participants with stroke of less than 2 weeks. The sample size had 90% power to detect changes in the outcomes measured (pain, muscle tone and shoulder subluxation). These authors established that even though not statistically significant, longitudinal shoulder strapping was more effective than circumferential in managing shoulder subluxation (0.74) and pain (*p* = 0.92). The treatment is feasible because the shoulders were strapped for 2 weeks, with the strapping being changed every 3–4 days; however, it may be difficult for some patients to come to the hospital twice a week following discharge because of the accessibility challenges mentioned earlier in this article (Rhoda et al. 2015). Van Wyk (2016) did a randomised controlled trial in the Free State Province to test effectiveness of shoulder stability exercises using the Biodex Balance System (Biodex Medical System 2011) in addition to usual upper limb rehabilitation compared with usual upper limb rehabilitation only. The intervention was not effective, but this is attributed to the small sample size of 17 stroke survivors. The intervention was feasible for a research project and for inpatients. Outside the research setting, it may be difficult for outpatients to come to the hospital 3 times per week for 28 sessions. Another limiting factor is that most rehabilitation centres do not have a Biodex. However, further research with a larger sample size is recommended, and if possible, also comparison of Biodex with shoulder stabilisation exercises that do not require expensive equipment for relevance in a resource poor setting.

#### Balance and stability

It is important to improve balance. An observational study conducted in Johannesburg by Muller (2015), of 40 stroke survivors, demonstrated a strong correlation between balance and functioning in activities of daily living. Balance was tested using the Berg Balance Scale and functional ability with the Functional Independence Measure. These are valid and reliable outcome measures, and thus the study findings are worth considering in clinical practice; however, they are only evidenced for use in the early stages of rehabilitation because participants were between 21 and 40 days after stroke.

Puckree and Naidoo (2014) undertook a study in KwaZulu-Natal comparing the effect of a balance and stability-focused outpatient community-based rehabilitation programme to a regular physiotherapy programme on outcomes of balance, stability and perceptions of improvement after acute stroke. Regular physiotherapy was 1-h individualised sessions fortnightly or group exercises for patients who were able to mobilise independently.

The experimental intervention included 12 sessions of exercises to improve stability and lower limb proprioception in sitting, standing and walking, performed fortnightly over a 7-month period with 25 stroke survivors. Assessment was done using valid and reliable outcome measures: Postural Assessment Scale for Stroke Patients, Berg Balance Scale and Barthel Index for stability, balance and functional ability, respectively. There was no significant difference between the control and intervention group at 7 months in any outcome; however, stability scores improved by 10%, balance scores by 14.6% and functional ability by 5% in the intervention group. An association between stability and balance (*r* = 0.84, *p* < 0.001) and between stability and functional ability (*r* = 0.74, *p* < 0.001) was found. This study showed a need to focus on stability in all three for balance and functional ability to improve. There is a need to repeat this study with a larger sample size and with outcome measures that may detect smaller changes in balance control such as functional reach or step test in addition to the global measures used.

#### Gait

Rehabilitation should include gait re-education to improve ambulation speed and endurance because some of the problems experienced by people after stroke are dependence with walking and stair climbing at discharge (Joseph & Rhoda 2013). Walking training can be done over ground or on a treadmill. Research in these areas has not yet been undertaken in South Africa, but the treadmill is used in SA clinical practice. A German study found low-to-moderate quality evidence of the rehabilitation benefits of treadmill training for stroke survivors who could already walk (Mehrholz, Thomas & Elsner 2017). An Australian study, however, established that treadmill walking with body weight support (BWS) improves walking speed for patients who cannot walk (Ada et al. 2010a). However, an American RCT established that locomotor training including use of body weight support on a treadmill was shown to be similar to progressive home exercises managed by a physiotherapist (Duncan et al. 2011).

Some patients may be anxious about treadmill training and thus should try walking over ground with a harness without BWS. Lines and footprints on the floor can be used to control step length and width and metronomes can be used to improve walking rhythm and timing (Ada et al. 2010b). Most stroke rehabilitation facilities in South Africa have a standard treadmill, but most of them do not have a harness for BWS.

Besides walking, gait can be improved by focusing on muscle strengthening of lower limb muscles. Gait pattern, speed and endurance may improve if the following muscles are targeted for strengthening: ankle dorsiflexors, hip flexors, ankle evertors, knee flexors and hip internal rotators (Dorsch et al. 2012). This Australian study by Dorsch et al. (2012) was conducted on 60 stroke survivors who were in the chronic stage (1–6 years after stroke), and thus the findings cannot be applied to patients in the acute and subacute phase. In this study, the combined strength of the ankle dorsiflexors and the hip flexors accounted for 34% of the variance in walking speed (*p* < 0.001) and the ankle dorsiflexors accounted for 31% of the variance (*p* < 0.001). Thus, these muscles have to be strengthened to improve gait.

Knox et al. (2018), in their randomised control trial of 144 stroke survivors, found that task-orientated circuit gait training improves gait speed and velocity in people 9.5 weeks after stroke, who could walk 10 m at less than 1.1 m/s without assistance. Knox et al.’s (2018) study consisted of an intervention of six 1-h sessions of task-orientated circuit gait training with a caregiver over a 12-week period for stroke survivors in the SA public health sector. The study compared three groups: (1) a group who did task-orientated circuit gait training (to improve strength, balance and task performance while standing and walking) facilitated by a caregiver; (2) a group who received strength training of lower extremities in sitting and lying and (3) a control group who received one 90-min educational session on stroke management. The task-orientated circuit-training programme with caregiver help improved gait recovery and walking competency after stroke (*p*-values between < 0.001 and 0.04). The following outcome measures were used in this study: Berg Balance Scale, 6MWT, the 10-m gait speed tests and the Timed Up and Go test. This is an example of an intervention that accommodates the needs of resource poor facilities and communities. The sessions did not require complex equipment and were provided over a 12-week period giving patients an opportunity to come once every 2 weeks if necessary for the six sessions.

#### Unilateral spatial neglect

Unilateral spatial neglect affects a person’s ability to perform activities of daily living. Van Wyk, Eksteen and Rheeder (2014) did a randomised control trial in Pretoria to determine the effect of saccadic eye movement training with visual scanning exercises (VSEs) integrated with task-specific activities on unilateral spatial neglect post-stroke. All 24 stroke survivors who had unilateral spatial neglect following a clinical ischaemic or haemorrhagic stroke within 1 and 3 weeks received task-specific activities 5 days a week for 4 or 5 weeks. Visual scanning exercises improve visual scanning behaviour by encouraging patients with neglect to actively pay attention to stimuli on the affected side, usually by starting a few degrees past midline, and going out from there. The intervention group (*n* = 12) did saccadic eye movement training with VSEs integrated with task-specific activities. Significant differences were noted on the King-Devick Test (*p* = 0.021), Star Cancellation Test (*p* = 0.016) and Barthel Index (*p* = 0.004). Thus, visual perceptual processing of patients who received VSEs improved, and this translated into better visual function and ability to perform activities of daily living following the stroke. This intervention is feasible for inpatients but not for outpatients if it is difficult for them to come to the hospital 5 days a week for 4 consecutive weeks. The researchers noted this limitation and are in the process of testing this intervention within a community setting with reduced frequency of intervention sessions.

#### Home exercise programme

It may not be possible for therapists to supervise all rehabilitation, especially in the home environment, and so unsupervised home-based exercise programmes are frequently prescribed to patients on discharge. For these exercises to be effective, they need to be performed at the appropriate dose; adherence to prescribed exercise programmes is therefore important. Kara and Ntsiea (2015) undertook a study in Gauteng to determine the effect on adherence of a written and pictorially presented prescription of a home exercise programme compared to verbal prescription in individuals with stroke. The control group (*n* = 21) received a home exercise programme with verbal instructions, and the intervention group (*n* = 21) received the same but with additional written and pictorial instructions for the exercises. Adherence was measured with an exercise logbook, which contained a column with exercises prescribed and a column for the patient and caregiver to tick that exercise was done and a third column to tick time that exercise was started and completed (exercise duration). The addition of a written and pictorial home exercise prescription did not result in better adherence to the home exercise programme. The use of a logbook served as reminder and track record for both groups, and thus in this case, monitoring with a logbook was more effective than the type of home exercise prescription. Adherence is a complex, multifactorial entity and does not only relate to the stroke survivor. Adherence also involves a change of behaviour, so while a person may be willing to follow prescribed instructions, they may not actually carry out the prescribed activity or exercise (Schneiders, Zusman & Singer 1998).

Caregiver education and prescription of home exercise programmes must take into consideration patient and contextual factors. In Gauteng, Mudzi et al. (2012b) conducted a randomised controlled trial to test the effectiveness of a caregiver education programme. This study compared the standard existing rehabilitation stroke care that was offered at the hospital at the time of the study (did not include individualised hands-on training of caregivers) and rehabilitation that included training in lifting and handling techniques, back care, continence, assistance with activities of daily living and communication. The intervention also included information on stroke-related problems and their management. This robust, fully powered study established that carer education alone did not result in significant improvements in patients’ functional abilities. The authors suggest that the reasons for lack of clinically significant changes in the two groups are that most of the patients (70%) did not have rehabilitation post-discharge and that they had poor functional ability at discharge from the hospital.

Therefore, there is a need to investigate ways to improve the way home exercise programmes are delivered and supported to improve post-discharge rehabilitation. Caregivers have a lot of responsibilities and they experience emotional strain and burden. This may influence stroke survivors’ adherence to home exercise programmes and recovery of function as some of them rely upon caregivers for assistance (Scorrano, Ntsiea & Maleka 2018). Thus, caregivers and stroke survivors need more support post-discharge.

#### Return to work intervention

According to the Health Professions Council of South Africa (HPCSA), rehabilitation includes the following: getting the patient to maximum potential in both work and sport, and community care includes offering services at industries and other organisations (HPCSA, scope of the profession of physiotherapy). The World Confederation for Physical Therapy (WCPT) also acknowledges that rehabilitation services may include assessment of ability to assume or resume work, ability to gain access to work and safety in work (WCPT entry level education guideline 2011). The WCPT also acknowledges that rehabilitation can be offered within a variety of settings, including (but not limited to) industrial and occupational settings (WCPT Education Policy Statement 2011). Thus, the HPCSA and WCPT include the role of physiotherapists in return to work (RTW) interventions. Just like intervention at all levels of care, RTW intervention also requires a multidisciplinary approach that includes physiotherapy (Ntsiea et al. 2015).

Ntsiea et al. (2015) conducted a multidisciplinary randomised controlled trial in Gauteng to determine the effect of a workplace intervention programme on the rate of RTW of stroke survivors who were working before the stroke (*n* = 40) compared with a control group (*n* = 40) who received usual care (general activities to improve impairments and activity limitations and prepare stroke survivors for RTW but without doing workplace visits). The workplace intervention programme was tailored according to each stroke survivor’s functional ability and workplace challenges. The workplace intervention consisting of workability assessments and six workplace visits was found to increase the odds of returning to work by 5.2 compared to the control group. Stroke survivors who returned to work had better quality of life (mean difference = 9.7, *p* = 0.05). Given the limited number of therapists or resources in South Africa, it may not be possible to have six workplace visits. To increase the rate of RTW, regular and early communication between the stroke survivor, employer and therapist is necessary to make well-informed decisions about the RTW process, and to explore possibilities of reasonable accommodation at the workplace (Ntsiea & Van Aswegen 2017).

### General considerations

During rehabilitation of stroke survivors, therapists need to think of benefits of intensity of rehabilitation. Treatment sessions of 30–60 min, 5–7 days a week may provide a significant beneficial effect (Pollock et al. 2014). In a SA context, this may not be possible for most patients either because of shortages of therapists in the hospital or because of transport costs if attending an outpatient service. Thus, patients may benefit from a ‘Patient-Directed Activity Program’ (Tremmell et al. 2017:1), which includes ‘activity stations designed to increase repetition, stimulation, attention, and activity of the affected upper extremities, lower extremities, and trunk’ completed independently in the ward, in addition to standard of care. Patients can do the activities instead of being inactive in their wards or homes and this may contribute towards increasing the intensity of their rehabilitation.

This review has focused on interventions aimed at improving specific impairment, ability or participation limitations presenting post-stroke researched within a SA context. It is acknowledged, however, that a holistic approach is needed during stroke rehabilitation, and this should include:

Provision of assistive devices, structural changes to houses, yards, roads and buildings where necessary, lobbying for accessible, affordable public transport, access audits of public buildings, and inclusion of non-governmental organisations and home-based care services in a seamless network of care, and thus requires an inter-professional model of care. (Cawood & Visagie 2015:8)

## Conclusion

Most of the stroke-related research in South Africa focused more on description of problems experienced; however, more intervention studies are now underway to develop context-specific interventions with feasible treatment intensity, frequencies and equipment requirements. Some studies have been undertaken and reported, providing slight evidence for the use of balance re-education exercises in the community; moderate evidence for the use of longitudinal shoulder strapping, VSE for unilateral spatial hemineglect; and strong evidence for gait re-education using task-orientated circuit exercise, and workplace intervention programme to increase return to work after stroke. Caregiver education alone and use of pictorial exercise programmes do not improve patients’ functional ability and adherence to home exercise programmes. This review also identified that all core outcome measures on Kwakkel et al.’s (2017) list were used in stroke rehabilitation studies in South Africa except for the Action Research Arm test and the 10-m walk test.

This review provides guidance to SA physiotherapists working in stroke rehabilitation as to the evidence for interventions evaluated in a SA context or interventions evaluated internationally deemed by the author to be appropriate for SA. Future research should explore new ways of improving post-discharge rehabilitation services. Examples of intervention studies that may be beneficial in a SA context are mirror therapy, mental practice and patient-directed activities in rehabilitation.

## Limitations

Some of the unpublished SA research reports and theses may have been missed when searching for literature if not loaded on a publicly available library repository.

## Footnotes

1 A simple hut built from tin, wood, or other materials (Collins dictionary).

2 Community service therapist is a qualified therapist who has just graduated, who works for one year in government institutions as a compulsory service to the country before registering as an independent practitioner.

## References

[CIT0001] Ada L., Dean C.M., Morris M.E., Simpson J.M. & Katrak P., 2010a, ‘Randomized trial of treadmill walking with body weight support to establish walking in subacute stroke, the MOBILISE trial’, *Stroke* 41, 1237–1242. 10.1161/STROKEAHA.109.569483 20413741

[CIT0002] Ada L., Dean C.M., Varga J. & Ennis S., 2010b, ‘Mechanically assisted walking with body weight support results in more independent walking than assisted overground walking in non-ambulatory patients early after stroke: A systematic review’, *Journal of Physiotherapy* 56, 153–161. 10.1016/S1836-9553(10)70020-5 20795921

[CIT0003] Arowoiya A.I, Elloker T., Karachi F., Mlenzana N., Jacobs-Nzuzi Khuabi L. & Rhoda A., 2017, ‘Using the World Health Organization’s disability assessment schedule II to assess disability in community-dwelling stroke patients’, *South African Journal of Physiotherapy* 73, 1–7. 10.4102/sajp.v73i1.343 PMC6093092 30135900

[CIT0004] Bernhardt J., Hayward K.S., Kwakkel G., Ward N.S., Wolf S.L., Borschmann K. et al., 2017, ‘Agreed definitions and a shared vision for new standards in stroke recovery research: The stroke recovery and rehabilitation roundtable taskforce’, *International Journal of Stroke* 12(5), 444–450. 10.1177/1747493017711816 28697708

[CIT0005] Bertram M.Y., Katzenellenbogen J., Vos T., Bradshaw D. & Hofman K.J., 2013, ‘The disability adjusted life years due to stroke in South Africa in 2008’, *International Journal of Stroke* 80, 76–80. 10.1111/j.1747-4949.2012.00955.x 23295022

[CIT0006] Biodex Medical Systems, 2011, *Balance system sd* [Online], Biodex Medical Systems, New York, viewed n.d., from http://www.biodex.com/physical-medicine/products/balance/balance-system-sd.

[CIT0007] British Society of Rehabilitation Medicine, 2003, *Rehabilitation following acquired brain injury: National clinical guidelines*, Physicians Royal College, London, UK, viewed 22 June 2018, from https://www.headway.org.uk/media/3320/bsrm-rehabilitation-following-acquired-brain-injury.pdf.

[CIT0008] Bryer A., Connor M., Haug P., Cheyip B., Staub H., Tipping B. et al., 2010, ‘South African guideline for management of ischaemic stroke and transient ischaemic attack 2010: A guideline from the South African Stroke Society (SASS) and the SASS Writing Committee’, *South African Medical Journal* 100(11 Pt 2), 747–778. 10.7196/SAMJ.4422 21081029

[CIT0009] Cawood J. & Visagie S., 2015, ‘Environmental factors influencing participation of stroke survivors in a Western Cape setting’, *African Journal of Disability* 4, 1–9. 10.4102/ajod.v4i1.198 PMC5433486 28730037

[CIT0010] Comley-White N., Mudzi W. & Musenge E., 2018, ‘Effects of shoulder strapping in patients with stroke: A randomised control trial’, *South African Journal of Physiotherapy* 74(1), a430 10.4102/sajp.v74i1.430 PMC6131703 30214946

[CIT0011] De Villiers L., Badri M., Ferreira M. & Bryer A., 2011, ‘Stroke outcomes in a socio-economically disadvantaged urban community’, *South African Medical Journal* 101, 345–348. 10.7196/SAMJ.4588 21837880

[CIT0012] Dorsch S., Ada L., Canning C.G., Al-Zharani M. & Dean C., 2012, ‘The strength of the ankle dorsiflexors has a significant contribution to walking speed in people who can walk independently after stroke: An observational study’, *Archives of Physical Medicine and Rehabilitation* 93(6), 1072–1076. 10.1016/j.apmr.2012.01.005 22464738

[CIT0013] Duff N., Ntsiea M.V. & Mudzi W.M., 2014, ‘Factors that influence return to work after stroke’, *Occupational Health Southern Africa* 20(3), 6–12.

[CIT0014] Duncan P., Sullivan K., Behrman A., Azen S., Wu S.S., Nadeau S.E. et al., 2011, ‘Body-weight-supported treadmill rehabilitation after stroke’, *The new England Journal of Medicine* 364, 2026–2036. 10.1056/NEJMoa1010790 21612471 PMC3175688

[CIT0015] *Framework and strategy for disability and rehabilitation services in South Africa 2015–2020*, viewed 22 June 2018, from http://www.health.gov.za/index.php/2014-08-15-12-54-26/category/266-2016-str?download=1569:framework-and-strategy-final-print-ready-2016.

[CIT0016] Health Professions Council of South Africa, *Physiotherapy scope of practice*, viewed from https://www.hpcsa.co.za/Uploads/editor/UserFiles/downloads/rules_reg_constitution/scope_of_profession_of_physiotherapy.pdf.

[CIT0017] Hilton J., Mudzi W., Ntsiea M.V. & Olorunju S., 2013, ‘Caregiver strain and quality of life six to 36 months post stroke’, *South African Journal of Physiotherapy* 69(4), a382 10.4102/sajp.v69i4.382

[CIT0018] Joseph C. & Rhoda A., 2011, ‘Outcome measures used to assess disability post stroke within the framework of the ICF: A literature review’, *Journal of Community and Health Sciences* 6(1), 49–52.

[CIT0019] Joseph C. & Rhoda A., 2013, ‘Activity limitations and factors influencing functional outcome of patients with stroke following rehabilitation at a specialised facility in the Western Cape’, *African Health Sciences* 13(3), 646–654. 10.4314/ahs.v13i3.18 24250302 PMC3824462

[CIT0020] Kara S. & Ntsiea M.V., 2015, ‘The effect of a written and pictorial home exercise prescription on adherence for people with stroke’, *Hong Kong Journal of Occupational Therapy* 26, 33–41. 10.1016/j.hkjot.2015.12.004

[CIT0021] Knox M., Stewart A. & Richards C.L., 2018, ‘Six hours of task-oriented training optimizes walking competency post stroke: A randomized controlled trial in the public health-care system of South Africa’, *Clinical Rehabilitation* 32(8), 1057–1068. 10.1177/0269215518763969 29529870

[CIT0022] Krakauer J.W., Carmichael S.T., Corbett D. & Wittenberg G.F., 2012, ‘Getting neurorehabilitation right: What can be learned from animal models?’, *Neurorehabilitation and Neural Repair* 26, 923–931. 10.1177/1545968312440745 22466792 PMC4554531

[CIT0023] Kusambiza-Kiingi A., Maleka D. & Ntsiea V., 2017, ‘Community reintegration and satisfaction of survivors of stroke receiving physiotherapy services in the community health centres within the Johannesburg area’, *African Journal of Disability* 6, 1–8. 10.4102/ajod.v6i0.296 PMC5502467 28730068

[CIT0024] Kwakkel G., Lannin N.A., Borschmann K., English C., Ali M., Churilov L. et al., 2017, ‘Standardized measurement of sensorimotor recovery in stroke trials: Consensus-based core recommendations from the stroke recovery and rehabilitation roundtable’, *International Journal of Stroke* 12(5), 451–461. 10.1177/1747493017711813 28697709

[CIT0025] Mabunda S., London L. & Pienaar D., 2017, ‘An evaluation of the role of an intermediate care facility in the continuum of care in Western Cape, South Africa’, *International Journal of Health Policy and Management* 7(2), 167–179. 10.15171/ijhpm.2017.52 29524940 PMC5819376

[CIT0026] Maleka M., Stewart A.S. & Hale L., 2017, ‘The development of a community reintegration outcome measure to assess people with stroke living in low socioeconomic areas’, *Edorium Journal of Disability and Rehabilitation* 3, 11–24.

[CIT0027] Mamabolo M.V., Mudzi W., Stewart A., Olorunju S. & Singh A., 2009, ‘A study to determine post discharge functional improvements in patients with stroke’, *South African Journal of Occupational Therapy* 39(1), 15–18.

[CIT0028] Mapipa H.V., Wolvaardt J.E. & Senkubuge F., 2016, ‘Adherence to rehabilitative programmes by patients living with neurological conditions: A South African context’, *African Journal for Physical Activity and Health Sciences* 22, 1157–1169.

[CIT0029] Maredza M., Bertram M.Y. & Tollman S.M., 2015, ‘Disease burden of stroke in rural South Africa: An estimate of incidence, mortality and disability adjusted life years’, *BMC Neurology* 15, 54 10.1186/s12883-015-0311-7 25880843 PMC4396076

[CIT0030] Masuku K.P., Mophosho M. & Tshabalala M., 2017, ‘“I felt pain. Deep pain…”: Experiences of primary caregivers of stroke survivors with aphasia in a South African township’, *African Journal of Disability* 7, 1–7. 10.4102/ajod.v7i0.368 PMC5843930 29535917

[CIT0031] Mehrholz J., Thomas S. & Elsner B., 2017, ‘Treadmill training and body weight support for walking after stroke’, *Cochrane Database of Systematic Reviews* (1), CD002840 10.1002/14651858.CD002840.pub4 28815562 PMC6483714

[CIT0032] Mji G., Chappell P., Statham S., Mlenzana N., Goliath C., De Wet C. et al., 2013, ‘Discourse of rehabilitation: With reference to disability models and rehabilitation policies for evaluation research in the South African setting’, *South African Journal of Physiotherapy* 69(2), 4–9. 10.4102/sajp.v69i2.22

[CIT0033] Muller C., 2015, *A study to determine which motor deficit has the strongest association with an improvement in functioning in activities of daily living in stroke patients*, Unpublished Research Report, University of the Witwatersrand, viewed 20 June 2018, from http://hdl.handle.net/10539/18491.

[CIT0034] Mudzi W., Stewart A. & Musenge E., 2012a, ‘Case fatality of patients with stroke over a 12-month period post stroke’, *The South African Medical Journal* 102(9), 756–767. 10.7196/SAMJ.5742 22958702

[CIT0035] Mudzi W., Stewart A. & Musenge E., 2012b, ‘Effect of carer education on functional abilities of patients with stroke’, *International Journal of Therapy and Rehabilitation* 19(7), 380–385. 10.12968/ijtr.2012.19.7.380

[CIT0036] Mudzi W., Stewart A. & Musenge E., 2013, ‘Community participation of patients 12 months post-stroke in Johannesburg South Africa’, *African Journal of Primary Health Care and Family Medicine* 5(1), 426–429. 10.4102/phcfm.v5i1.426

[CIT0037] National Rehabilitation Policy, 2000, *Rehabilitation for all. Republic of South Africa*, Department of Health, viewed 18 June 2018, from file:///C:/Users/00100536/Downloads/national_rehabilitation_policy.pdf.

[CIT0038] Ntsiea M.V. & Van Aswegen H., 2017, ‘Barriers to and enablers of return to work after stroke: Survivor and employer perceptions’, *Occupational Health Southern Africa* 23(2), 23–30. 10.1177/0269215514554241

[CIT0039] Ntsiea M.V., Van Aswegen H., Lord S. & Olorunju S., 2015, ‘The effect of a workplace intervention programme on return to work after stroke: A randomised controlled trial’, *Clinical Rehabilitation* 29(7), 663–673. 25322870 10.1177/0269215514554241

[CIT0040] Patterson S.L., 2018, *Return to work after stroke, rate, facilitators and barriers in Buffalo City, South Africa*, Unpublished research report, Stellenbosch Witwatersrand, viewed 25 December 2018, from https://scholar.sun.za.

[CIT0041] Pollock A., Farmer S.E., Brady M.C., Langhorne P., Mead G.E., Mehrholz J. et al., 2014, ‘Interventions for improving upper limb function after stroke’, *Cochrane Database of Systematic Reviews* (11), CD010820 10.1002/14651858.CD010820.pub2 25387001 PMC6469541

[CIT0042] Puckree T. & Naidoo P., 2014, ‘Balance and stability-focused exercise program improves stability and balance in patients after acute stroke in a resource-poor setting’, *PM&R* 6(12), 1081–1087. 10.1016/j.pmrj.2014.06.008 24982002

[CIT0043] Rhoda A., Cunningham N., Azaria S. & Urimubenshi G., 2015, ‘Provision of inpatient rehabilitation and challenges experienced with participation post discharge: Quantitative and qualitative inquiry of African stroke patients’, *BMC Health Services Research* 15, 423 10.1186/s12913-015-1057-z 26412081 PMC4584463

[CIT0044] Rhoda A., Mpofu R. & De Weerdt W., 2009, ‘The rehabilitation of stroke patients at community health centres in the Western Cape’, *South African Journal of Physiotherapy* 65(30), 1–6. 10.4102/sajp.v65i3.87

[CIT0045] Rhoda A., Mpofu R. & De Weerdt W., 2011, ‘Activity limitations of patients with stroke attending out-patient facilities in the Western Cape, South Africa’, *South African Journal of Physiotherapy* 67(2), 16–22. 10.4102/sajp.v67i2.41

[CIT0046] Rhoda A., Smith M., Putman K., Mpofu R., De Weerdt W. & De Wit L., 2014, ‘Motor and functional recovery after stroke: A comparison between rehabilitation settings in a developed versus a developing country’, *BMC Health Services Research* 14, 82 10.1186/1472-6963-14-82 24559193 PMC3974037

[CIT0047] Rhoda A.J., 2014, ‘Health-related quality of life of patients six months poststroke living in the Western Cape, South Africa’, *African Journal of Disability* 3(1), 126 10.4102/ajod.v3i1.126 28730004 PMC5443046

[CIT0048] Schneiders A.G., Zusman M. & Singer K.E., 1998, ‘Exercise therapy compliance in acute low back pain patients’, *Manual Therapy* 3, 147–152. 10.1016/S1356-689X(98)80005-2

[CIT0049] Scorrano M., Ntsiea V. & Maleka D., 2018, ‘Enablers and barriers of adherence to home exercise programmes after stroke: Caregiver perceptions’, *International Journal of Therapy and Rehabilitation* 25(7), 353–364. 10.12968/ijtr.2018.25.7.353

[CIT0050] Stroke Unit Trialists’ Collaboration, 2013, ‘Organised inpatient (stroke unit) care for stroke’, *Cochrane Database of Systematic Reviews* (9), CD000197 10.1002/14651858.CD000197.pub3 24026639 PMC6474318

[CIT0051] Titus A.W., Hillier S., Louw Q.A. & Inglis-Jassiem G., 2018, ‘An analysis of trunk kinematics and gait parameters in people with stroke’, *African Journal of Disability* 7, 1–6. 10.4102/ajod.v7i0.310 PMC5913771 29707514

[CIT0052] Trammell M., Kapoor P., Swank C. & Driver S., 2017, ‘Improving practice with integration of patient directed activity during inpatient rehabilitation’, *Clinical Rehabilitation* 31(1), 3–10. 10.1177/0269215515625100 26837432

[CIT0053] Van Wyk A., Eksteen C.A. & Rheeder P., 2014, ‘The effect of visual scanning exercises integrated into physiotherapy in patients with unilateral spatial neglect poststroke: A matched-pair randomized control trial’, *Neurorehabil Neural Repair* 28(9), 856–873. 10.1177/1545968314526306 24633138

[CIT0054] Van Wyk H., 2016, *The effect of shoulder stability training on upper limb function and quality of life in patients with hemiplegia*, Unpublished research report, University of the Witwatersrand, viewed 25 June 2018, from http://hdl.handle.net/10539/22574.

[CIT0055] Westaway M.S., 2010, ‘Effects of ageing, chronic disease and co-morbidity on the health and well-being of older residents of Greater Tshwane’, *South African Medical Journal* 100, 35–36. 20429485

[CIT0056] Wolf T., Baum C. & Connor L., 2009, ‘Changing face of stroke: Implications for occupational therapy practice’, *American Journal of Occupational Therapy* 63(5), 621–625. 10.5014/ajot.63.5.621 19785261 PMC2862359

[CIT0057] World Confederation for Physical Therapy, ‘Guidelines for physical therapist professional entry level education’, in *Revised and published as a WCPT guideline at the 17th General Meeting of WCPT June 2011*, viewed 18 May 2018, from www.wcpt.org/guidelines/entry-level-education.

[CIT0058] World Confederation for Physical Therapy, ‘Policy statement: Education’, in *Revised and re-approved at the 17th WCPT General Meeting, June 2011*, viewed 18 May 2018, from www.wcpt.org/policy/ps-education.

[CIT0059] World Health Organization, 2010, *Community based rehabilitation guidelines*, viewed 20 June 2018, from http://www.who.int/disabilities/cbr/guidelines/en/.

[CIT0060] World Health Organization, 2001, *Classification of functioning, disability and health: ICF short version*, WHO Library Cataloguing-in-Publication Data, Geneva.

